# Innovative molecular targets for combatting metastasis in adrenocortical carcinoma

**DOI:** 10.3389/fendo.2025.1516467

**Published:** 2025-10-30

**Authors:** Jianqiu Kong, Xiong Chen, Xinxiang Fan, Shuogui Fang, Qihang Zhang, Long Zhang, Chenxiao Liao, Qingqing He, Weibin Xie, Yichun Xing, Junjiong Zheng

**Affiliations:** ^1^ Department of Urology, Sun Yat-Sen Memorial Hospital, Sun Yat-Sen University, Guangzhou, China; ^2^ Guangdong Provincial Key Laboratory of Malignant Tumor Epigenetics and Gene Regulation, Sun Yat-Sen Memorial Hospital, Sun Yat-Sen University, Guangzhou, China; ^3^ Department of Radiotherapy, Sun Yat-Sen Memorial Hospital, Sun Yat-Sen University, Guangzhou, China; ^4^ Department of Pathology, Sun Yat-Sen Memorial Hospital, Sun Yat-Sen University, Guangzhou, China; ^5^ Department of Gynecology, Sun Yat-Sen Memorial Hospital, Sun Yat-Sen University, Guangzhou, China

**Keywords:** adrenocortical carcinoma, metastasis, weighted gene co-expression network analysis, gene-set enrichment analysis, protein-protein interactions

## Abstract

**Background:**

Metastasis in adrenocortical carcinoma (ACC) presents a formidable clinical challenge, with limited insights into its underlying mechanisms and few effective treatment options. This study aimed to identify molecular markers associated with metastasis in adult ACC and to explore novel therapeutic approaches.

**Methods:**

RNA sequencing data from 74 adult ACC patients were analyzed using bioinformatics approaches, including weighted gene co-expression network analysis (WGCNA) and differential gene expression analysis. Functional pathway enrichment analyses were conducted to identify metastasis-associated genes and to explore key biological pathways contributing to metastatic progression. Candidate genes were selected based on their statistical significance, prognostic relevance, and involvement in metastatic processes.

**Results:**

Seven hub genes were identified as significantly associated with metastasis and poor prognosis in ACC patients. These genes were enriched in pathways critical to tumor progression, such as mismatch repair, nucleotide excision repair, and cell cycle regulation. High expression levels of these genes correlated with reduced overall survival. Importantly, potential therapeutic agents targeting these molecular drivers were identified, offering promising, lower-toxicity options for treating metastatic ACC.

**Conclusion:**

The molecular markers identified in this study offer valuable insights into the mechanisms underlying ACC metastasis and represent promising therapeutic targets, providing a foundation for developing improved treatment strategies in clinical practice.

## Introduction

Adrenocortical carcinoma (ACC) is a rare endocrine malignancy, with an incidence of 0.7–2 cases per million annually (1, 2). Despite complete surgical resection of early-stage tumors, ACC patients remain at high risk for relapse and metastasis (1, 3). The 5-year survival rate for ACC patients who undergo complete surgical resection is approximately 30% (4, 5). However, recent data, including our findings, indicate that survival rates in adult ACC may range from 40% to 60%, depending on individual prognostic factors (6). For patients with metastatic ACC, unfortunately, the 5-year survival rate remains under 15% (7–9). Approximately one-third of ACC patients present with locally advanced cancer or synchronous metastasis at diagnosis (10), and more than half will develop distant metastases even after complete resection of the primary tumor (5, 11). Most deaths due to ACC are directly associated with metastatic disease (12).

Current treatment options for metastatic ACC remain limited and demonstrate suboptimal efficacy. The only FDA-approved drug, mitotane, primarily used as adjuvant therapy, has shown inconsistent survival benefits and significant toxicity, including gastrointestinal and neurological side effects (13). Combination chemotherapy regimens, such as EDP-M (etoposide, doxorubicin, cisplatin, and mitotane), provide moderate response rates but are associated with substantial toxicity, limiting their long-term use (14). Furthermore, targeted therapies, including IGF1R inhibitors and mTOR inhibitors, as well as immune checkpoint inhibitors, have demonstrated limited clinical efficacy, with response rates typically below 10% (15, 16). These challenges underscore the urgent need for novel molecular targets and therapeutic strategies to improve outcomes for metastatic ACC patients.

To enhance understanding of ACC and improve patient survival, substantial research has focused on its molecular mechanisms. Key factors in ACC pathogenesis include overexpression of *IGF2*, constitutive activation of *WNT/β-catenin* signaling (1, 17), and inactivation of the *TP53/RB* pathway (18). Gara et al. found that metastatic ACC exhibits a higher mutation rate and greater tumor heterogeneity than primary ACC, highlighting the insufficiency of studying primary tumors alone to fully understand metastasis in ACC patients (12).

In recent decades, genome-wide association studies have transformed our understanding of cancer biology and treatment discovery. Weighted gene co-expression network analysis (WGCNA) is a powerful bioinformatics tool that constructs gene co-expression modules, which can be summarized by module eigengenes and intramodular hub genes (19). WGCNA enables exploration of network module structure, relationships between genes and modules, inter-module associations, and gene or module ranking with respect to a sample trait, ultimately generating testable hypotheses for independent data validation (19). WGCNA has been successfully applied to cancers such as lymphoma, prostate cancer, and colon cancer (20–23); however, its application to ACC metastasis remains unexplored.

In this study, we applied WGCNA to transcriptional profiling data from The Cancer Genome Atlas (TCGA) for ACC to identify hub genes associated with metastasis. Additionally, we used the Drug Gene Interaction Database (DGIdb, http://dgidb.org/) to identify potential therapeutic agents that may prevent or treat metastatic ACC, aiming to extend patient survival.

## Methods

### Data collection and processing

**Figure 1:**
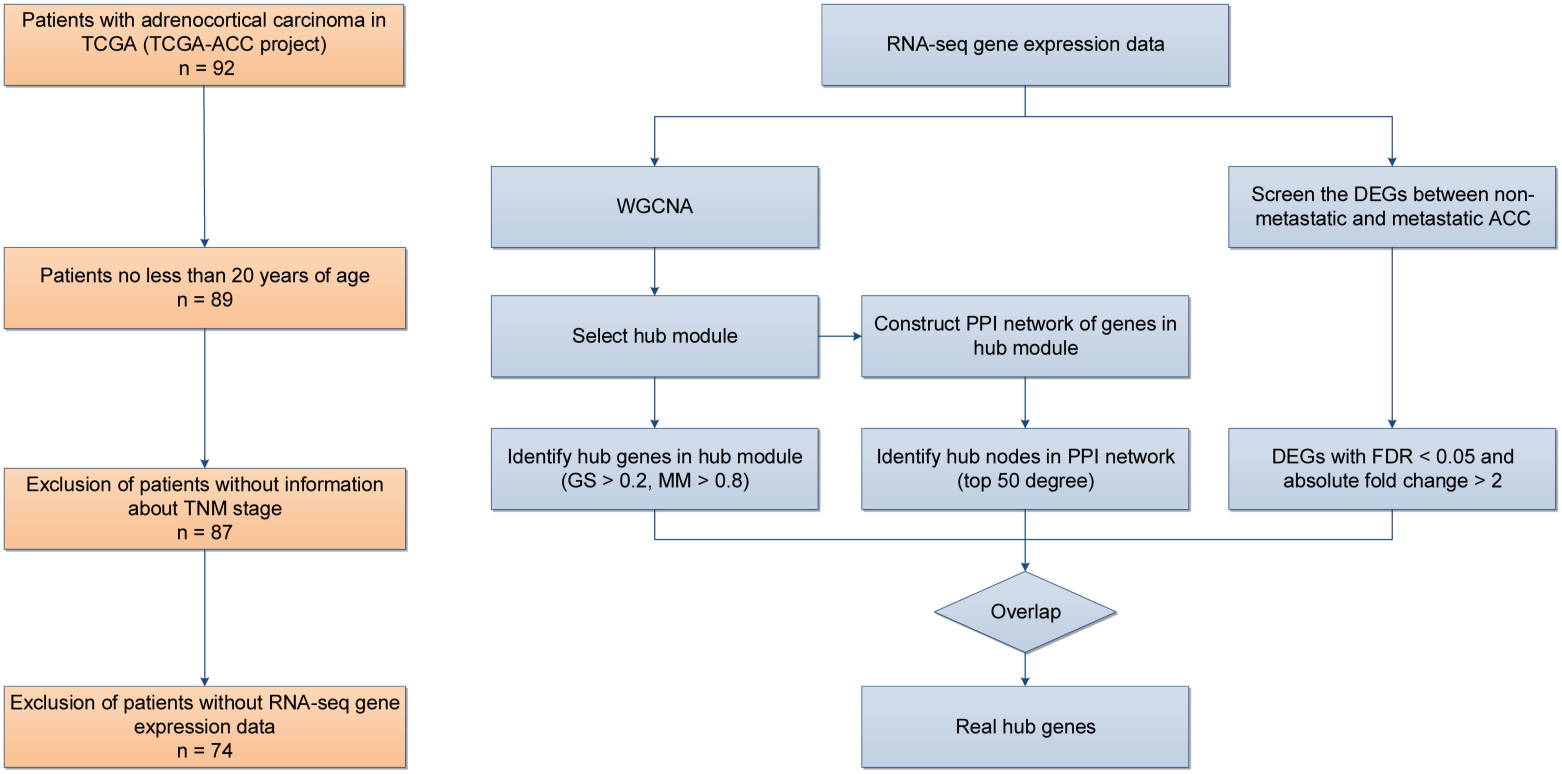
Cases selection process and study design.

Considering the distinct biological behavior of adult ACC compared to pediatric cases (24), only adult ACC patients from TCGA were included. Inclusion criteria were: (1) pathologically confirmed ACC; (2) age at diagnosis ≥ 20; (3) available data on metastasis stage; (4) available RNA-seq gene expression data. **Figure 1** illustrates the case selection process, which resulted in 74 cases for analysis. Level 3 RNA-seq data were obtained from the UCSC Cancer Genomics Browser (https://xenabrowser.net/), based on the Illumina platform, normalized and log2(FPKM+1) transformed. Gene symbols were standardized via Perl script, and duplicate gene expressions were averaged. After excluding low-expression genes, 16,115 genes were retained for further analysis. Clinicopathological and follow-up data were downloaded from TCGA (https://portal.gdc.cancer.gov/) on January 23, 2024.

### Weighted gene co-expression network analysis

The study design is shown in **Figure 1**. To ensure reliable WGCNA results, outlier samples distant from other samples were excluded. The “WGCNA” R package was used to analyze the top 25% of variant genes (4,029 genes) from the initial 16,115. An optimal power cut-off threshold was selected to create a scale-free topology overlap matrix (TOM), with average linkage hierarchical clustering applied to detect gene modules. Modules were visualized as dendrogram branches using the Dynamic Tree-Cut algorithm. Module eigengenes were calculated, and the module most highly correlated with the M stage was selected for further analysis. Gene significance (GS) and module membership (MM) were evaluated for genes within this module.

### Protein-protein interactions of the selected module

After identifying the most significant module via WGCNA, the STRING database (http://string-db.org/) was used to identify significant PPIs with an interaction score ≥ 0.7. The PPI network was visualized using Cytoscape software (http://cytoscape.org/).

### Identification of differentially expressed genes between metastasis and non-metastasis groups

DEGs were identified by comparing RNA-seq expression values between the metastasis and non-metastasis groups using the “edgeR” R package. The cut-off criteria for DEGs were FDR < 0.05 and a fold change (FC) > 2.0.

### Gene ontology and KEGG pathway enrichment analysis

GO enrichment analysis, covering biological processes (BP), cellular components (CC), and molecular functions (MF), and KEGG pathway enrichment analysis were conducted to understand the high-level functional implications of the gene clusters. Metascape (http://metascape.org/gp/index.html), a comprehensive annotation and analysis resource, was used to perform both GO and KEGG pathway enrichment analyses.

### Identification of hub genes

Hub genes were defined based on the following criteria: (1) genes within the selected module with MM > 0.8 and GS > 0.2; (2) degree > 20 in the PPI network; (3) DEGs between the metastasis and non-metastasis groups.

### Gene-set enrichment analysis

The “Survminer” R package was used to determine optimal cut-off values for hub gene expression levels. Patients were classified into high and low expression groups based on this threshold, and survival differences between groups were assessed using the log-rank test. GSEA software was used with the MSigDB reference gene set (c2.cp.kegg.v6.2.symbols.gmt) to identify enrichment pathways, with FDR < 0.05 as the cut-off. Visualization was conducted using the “plyr,” “grid,” and “ggplot2” R packages.

### Identification of targeted drugs for hub genes

The DGIdb database, which consolidates drug-gene interaction data, was consulted to identify FDA-approved drugs for each hub gene. The top 10 ranked drugs for each gene were recorded.

### Statistical analysis

All statistical analyses were performed using R version 3.5.1. Survival analysis was conducted with the “survival” R package, and a two-sided P < 0.05 was considered statistically significant.

## Results

### Demographic characteristics of the study cohort

After excluding one outlier sample through hierarchical clustering analysis, 73 cases were included in the analysis, consisting of 15 cases with metastatic disease (M1) and 58 without metastasis (M0). Among these patients, 57 (78.1%) were alive, and 16 (21.9%) had died by the last follow-up. The median follow-up duration was 3.2 years (interquartile range, 1.6–5.5 years). Additional demographic details are provided in **Table 1**.

**Table 1:** Baseline clinicopathological characteristics of the enrolled patients.

Variable	No. (%)
Age, years	
Median (interquartile range)	50 (36-60)
Sex	
Male	28 (38.4%)
Female	45 (61.6%)
Laterality	
Left	40 (54.8%)
Right	33 (45.2%)
T stage	
T1	8 (11.0%)
T2	39 (53.4%)
T3	8 (11.0%)
T4	18 (24.7%)
N stage	
N0	65 (89.0%)
N1	8 (11.0%)
M stage	
M0	58 (79.5%)
M1	15 (20.5%)
Survival status	
Alive	57 (78.1%)
Dead	16 (21.9%)
Follow-up time, years	
Median (interquartile range)	3.2 (1.6-5.5)

### WGCNA results

**Figure 2:**
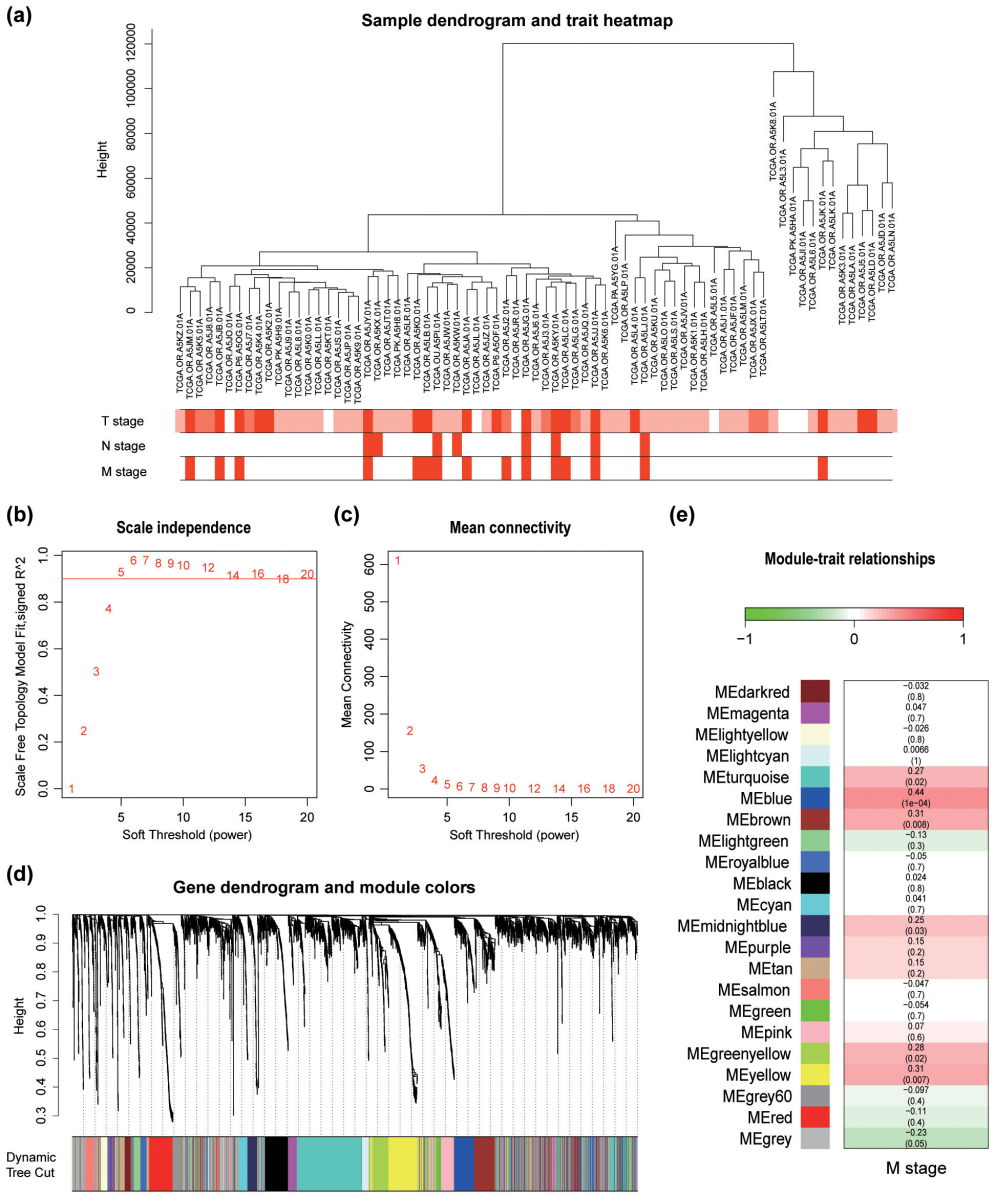
Weighted gene co-expression network analysis. **(a)** Clustering based on mRNA-seq data of 4,029 genes across 73 samples, with color intensity indicating T stage, N stage, and M stage. **(b)** Analysis of the scale-free index across various soft power thresholds. **(c)** Analysis of mean connectivity for different soft power thresholds. **(d)** Dendrogram of the 4,029 genes clustered using a dissimilarity measure (1-TOM). **(e)** Average gene significance and errors within modules associated with the M stage.

**Figure 3:**
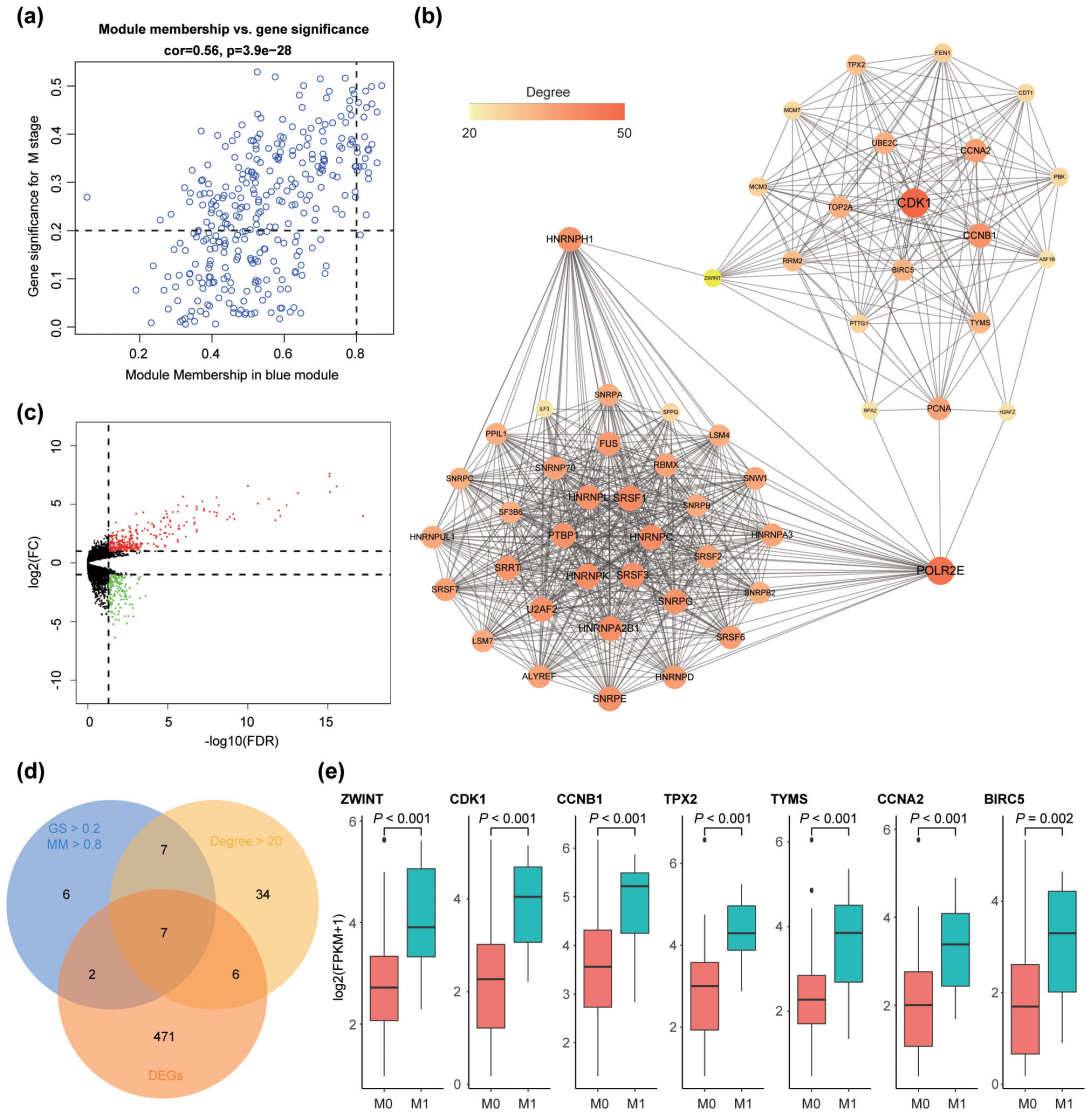
Identification of hub genes. **(a)** Scatter plot of module eigengenes (MEs) in the blue module. **(b)** Protein-protein interaction network for the blue module with nodes of degree > 20, where color intensity corresponds to node degree. **(c)** Volcano plot depicting differentially expressed genes (DEGs) in the metastasis group versus the non-metastasis group. Each point represents a gene probe; red indicates up-regulation, and green indicates down-regulation (adjusted FDR < 0.05, FC > 2.0). **(d)** Venn diagram showing overlapping genes that meet the hub gene selection criteria. **(e)** Expression levels of the seven hub genes in the metastasis versus non-metastasis groups.

A total of 4,029 genes (representing the top 25% of RNA-seq variants) were used to construct the gene co-expression network. A soft threshold power of 5 was selected, as it provided more than 90% similarity in topology models (**Figures 2b, c**), yielding 21 distinct modules (**Figure 2d**). Among these, the blue module exhibited the strongest correlation with metastasis (Pearson correlation coefficient = 0.44, *P*<0.001; **Figure 2e**). By setting criteria of GS > 0.2 and MM > 0.8, 22 candidate hub genes were identified and are visualized in **Figure 3a**.

### PPI network construction and identification of candidate hub genes

To further investigate the functional roles of genes within the selected module, a protein-protein interaction (PPI) network was constructed. Using an interaction score threshold of ≥ 0.7, a network with 217 nodes and 1,344 edges was generated. Nodes with a degree > 20 were visualized in **Figure 3b**, where *CDK1* exhibited the highest degree (degree = 50). Detailed information on the PPI network is provided in **Additional File 1: Table S1**.

### Differentially expressed genes between metastasis and non-metastasis groups

In total, 486 differentially expressed genes (DEGs) were identified, including 330 up-regulated and 156 down-regulated genes in the metastasis group compared to the non-metastasis group (**Figure 3c**). Among these, 30 DEGs were present within the selected module, including *TOP2A, ASF1B, EIF4EBP3, AC131097.2, TPX2, FEN1, TUBB2A, ZWINT, RRM2, TK1, CSDC2, MYBL2, CCNA2, NR0B1, STMN1, TYMS, CDT1, PTTG1, CDK1, RPS26P47, UBE2T, NUPR1, NDRG4, CTRB1, AIF1L, PKIG, APOBEC3B, BIRC5, UBE2S*, and *CCNB1*. Detailed DEG information is available in **Additional File 2: Table S2**.

### Biological characterization of selected module and DEGs: GO-BP and KEGG enrichment analysis

**Figure 4:**
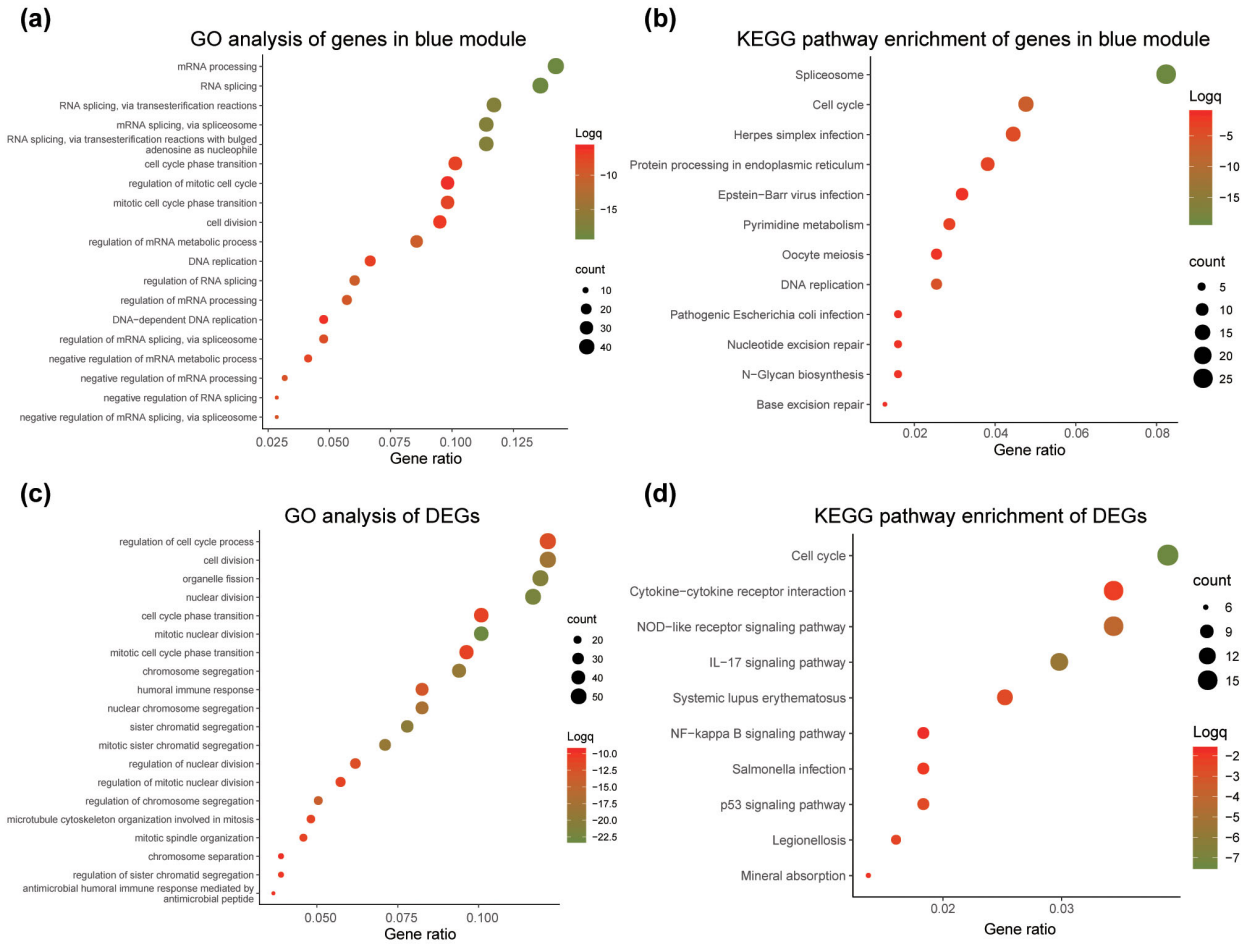
GO and KEGG pathway enrichment analysis for genes in the blue module and DEGs. The x-axis represents the gene ratio, and the y-axis lists the GO or KEGG pathway terms. The log10 (q-value) of each term is colored according to the legend, and dot sizes indicate the number of genes in each enriched term. **(a)** GO enrichment analysis of genes in the blue module. **(b)** KEGG pathway enrichment analysis of genes in the blue module. **(c)** GO enrichment analysis of DEGs. **(d)** KEGG pathway enrichment analysis of DEGs.

To gain insights into the biological roles of genes in the blue module and DEGs, gene annotation and enrichment analyses for Gene Ontology (GO-BP) and KEGG pathways were conducted. The significantly enriched GO-BP and KEGG pathway terms (*P <*0.05) for the blue module and DEGs are shown in **Additional File 3: Table S3**. **Figure 4a** highlights the top 19 GO-BP terms in the blue module, which include mRNA processing, RNA splicing, cell cycle phase transition, and mitotic cell cycle regulation, all of which are dysregulated in cancer. **Figure 4b** shows the top 12 enriched KEGG pathways for the blue module, including spliceosome, cell cycle, DNA replication, and nucleotide excision repair pathways. For DEGs, the top 20 enriched GO-BP terms included processes such as mitotic nuclear division, organelle fission, and chromosome segregation (**Figure 4c**), while the top 10 enriched KEGG pathways encompassed cell cycle, IL-17 signaling, and p53 signaling pathways (**Figure 4d**).

### Hub gene analysis

**Figure 5:**
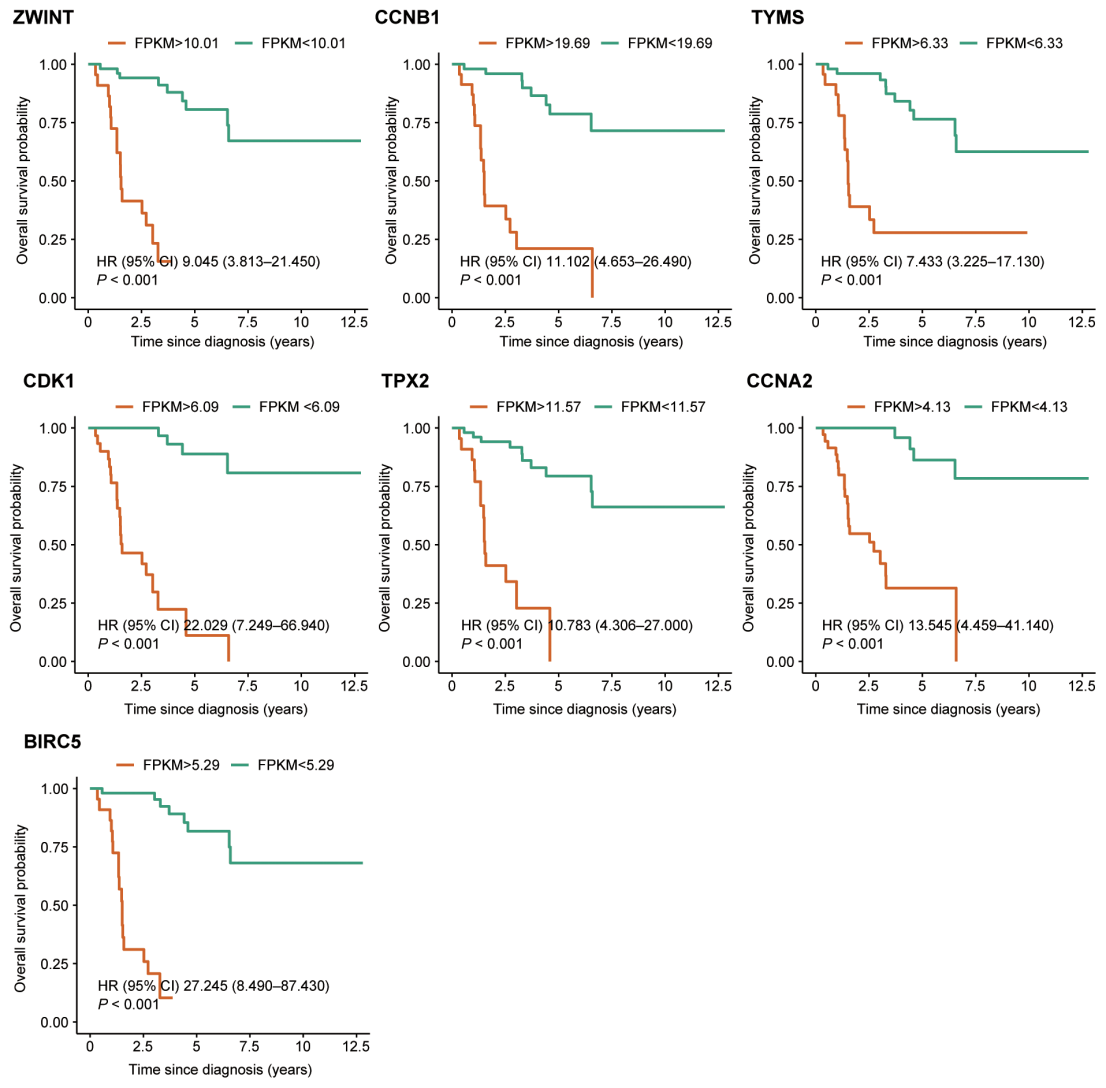
Kaplan–Meier survival analysis of the seven hub genes. Patients were grouped into high-expression and low-expression categories based on pre-defined cut-off values.

**Figure 6:**
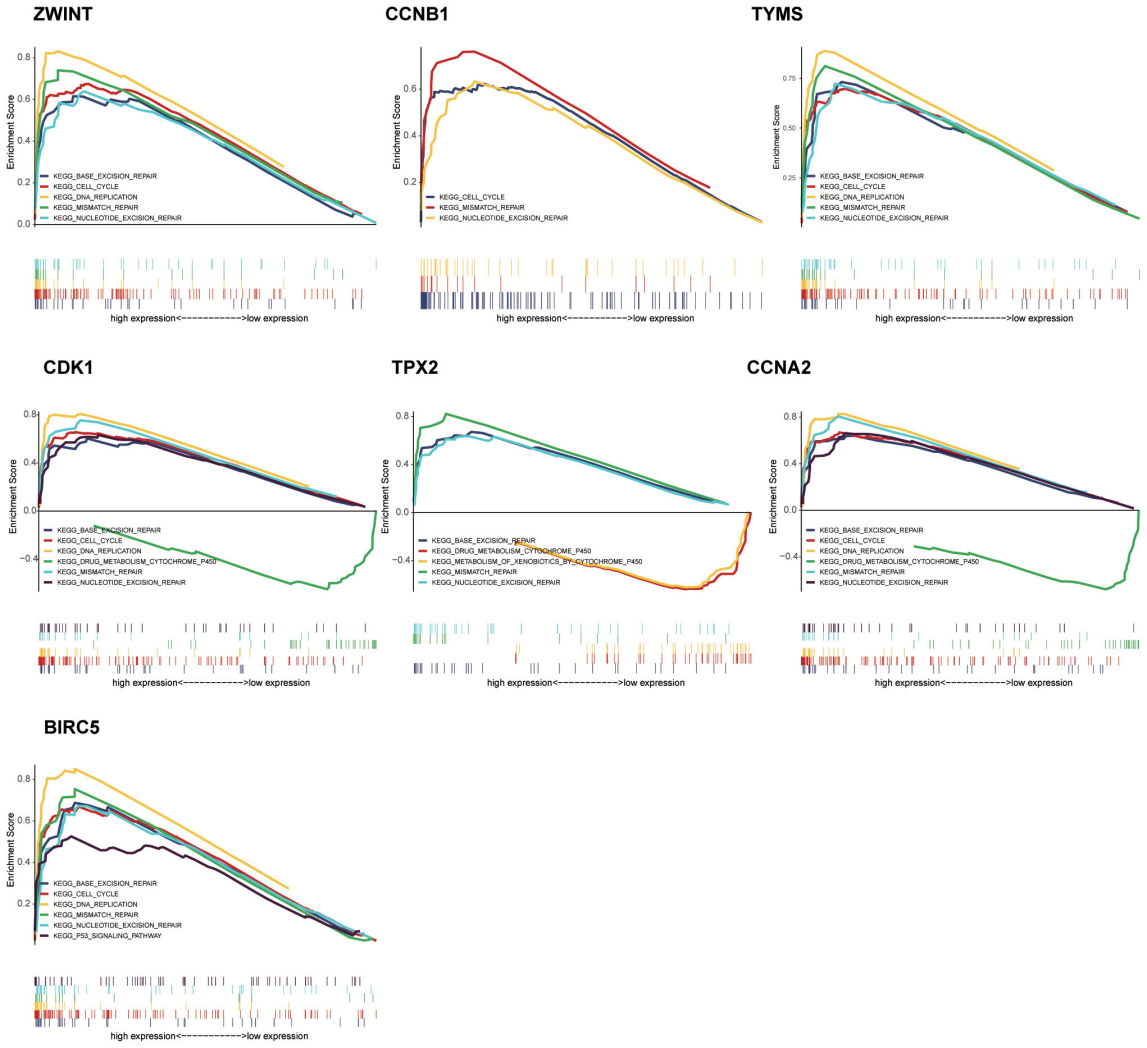
Gene set enrichment analysis of the seven hub genes (high versus low expression).

Based on the criteria defined in the *Methods* section, seven hub genes were identified: *ZWINT, CDK1, CCNB1, TPX2, TYMS, CCNA2*, and *BIRC5* (**Figure 3d**). All seven genes were significantly upregulated in the metastasis group (**Figure 3e**). Survival analysis and GSEA based on the optimal expression cut-off values revealed that high expression of these hub genes significantly correlated with reduced overall survival (*P <*0.001, **Figure 5**). Cox regression analysis further confirmed that CDK1 (HR 95%CI: 10.031 [2.499–40.260]) and BIRC5 (HR 95%CI: 5.215 [1.395–19.510]) were independent prognostic factors for poor overall survival in ACC patients. **Figure 6** presents the GSEA enrichment results for the hub genes, showing that high expression of these genes is enriched in pathways such as mismatch repair and nucleotide excision repair. Additionally, high expression of *ZWINT, TYMS, CDK1, CCNA2*, and *BIRC5* was associated with cell cycle and DNA replication pathways, while high expression of *ZWINT, CCNB1, TYMS, CDK1, TPX2, CCNA2*, and *BIRC5* was linked to mismatch repair and nucleotide excision repair. Notably, low expression of *TPX2* correlated with xenobiotic metabolism by cytochrome P450, whereas high expression of *BIRC5* was enriched in the p53 signaling pathway. Additional GSEA enrichment terms are provided in **Additional File 4: Table S4**.

### Targeted drugs for hub genes

Using the DGIdb, targeting drugs were identified for four of the hub genes: *CDK1, CCNA2, BIRC5*, and *TYMS*. These drugs, classified into categories such as hormones, nonsteroidal anti-inflammatory drugs (NSAIDs), tyrosine kinase inhibitors (TKIs), monoclonal antibodies, antibiotics, and others, are listed in **Table 2**.

**Table 2:** Targeting drugs of hub genes.

Gene	Targeting drugs	Category
*CDK1*	Eltrombopag, Romiplostim	Others
*CCNA2*	Ethinyl estradiol	Hormone
*BIRC5*	Trastuzumab	Monoclonal antibody
	Paclitaxel, Irinotecan, Doxorubicin, Cytarabine	Chemotherapy drug
	Sulindac	NSAID
	Lapatinib	TKI
	Plicamycin	Antibiotics
	Tretinoin, Arsenic trioxide, Interferon beta-1a	Others
*TYMS*	Trifluridine, Raltitrexed, Fluorouracil, Capecitabine, Floxuridine, Pemetrexed, Gemcitabine, Irinotecan	Chemotherapy drug
	Trimethoprim	Antibiotics
	Dexamethasone	Hormone

NSAID, Nonsteroidal anti-inflammatory drug; TKI, tyrosine kinase inhibitor.

## Discussion

In this study, we applied WGCNA to analyze ACC transcriptional data from TCGA, successfully identifying seven hub genes—*ZWINT, CDK1, TPX2, TYMS, CCNA2, CCNB1*, and *BIRC5*—closely associated with metastasis in adult ACC. These genes were significantly upregulated in the metastatic group, and survival analysis confirmed their association with patient prognosis. Further, using the DGIdb, we identified potential drugs targeting these hub genes, offering promising insights for therapeutic interventions.

ACC treatment options, especially for metastatic disease, remain limited. Epidemiological data show minimal improvement in survival outcomes for ACC patients from 1993–1998 to 2005–2010 (8). The sole FDA-approved drug, mitotane, has demonstrated uncertain benefits regarding overall survival (13). Other second- and third-line therapies for metastatic ACC also provide limited benefits (25). Despite the efficacy of PD-L1 antibodies in many cancers, a phase Ib trial in 50 metastatic ACC patients yielded only a 6% response rate (3 partial responses) (15). Similar limitations are observed with IGF1R-mTOR inhibitors (3.9% response rate) (3), tyrosine kinase inhibitors (TKIs, 1.4%) (3), and gemcitabine-based chemotherapy (4.9%) (16). Thus, discovering new molecular targets and more effective drugs for metastatic ACC is essential. Our study, by identifying key genes and possible drug targets for metastatic ACC, offers insights that may enhance patient outcomes.

The seven hub genes identified here contribute to several processes crucial for malignancy, including cell cycle progression, DNA repair, and replication fidelity. Based on GSEA results, these genes are implicated in malignant transformation pathways, such as cell cycle regulation, mismatch repair, DNA replication, and nucleotide excision repair. Using Gene Expression Profiling Interactive Analysis (GEPIA) and the UniProt knowledgebase, we investigated each hub gene’s function. We found that *CDK1* and *CCNA2* are essential regulators of the G1/S and G2/M cell cycle transitions, while *CCNB1* controls the G2/M checkpoint. *ZWINT* encodes a protein crucial for kinetochore function, and *TPX2* is involved in spindle microtubule organization during mitosis, being exclusively expressed in proliferating cells. These five genes are closely tied to cell cycle regulation and mitosis, aligning with known observations that Ki-67, a cell proliferation marker, is highly expressed in metastatic ACC (26, 27). *TYMS* encodes an enzyme that has been widely studied as a target for cancer chemotherapy, particularly as the primary site of action for drugs like 5-fluorouracil (5-FU) and folate analogs. Overexpression of *TYMS* is associated with resistance to 5-FU and poor prognosis in colorectal, breast, and other cancers (28). Given that mitotane is currently the only approved drug for metastatic ACC and its mechanism remains unclear, the overexpression of *TYMS* in metastatic ACC may potentially impact the efficacy of mitotane and other chemotherapies, a hypothesis warranting further investigation.

*BIRC5* has been shown to regulate key pathways involved in EMT, including Wnt/β-catenin and TGF-β signaling, which contribute to metastatic progression in ovarian and lung cancers. Its role in promoting apoptosis resistance and cell survival has also been implicated in pancreatic and nasopharyngeal cancers (29–31). By stabilizing β-catenin and enhancing TGF-β signaling, *BIRC5* facilitates the loss of epithelial markers such as E-cadherin and the gain of mesenchymal markers such as vimentin, thereby promoting cellular plasticity and migration. These mechanisms, extensively studied in ovarian and lung cancers, suggest a similar role for *BIRC5* in ACC metastasis. Our study confirms that high expression of BIRC5 is significantly associated with poor prognosis in ACC patients, consistent with its role in promoting metastasis and apoptosis resistance in other cancer types. Future studies should focus on validating these pathways in ACC using functional assays, such as gene knockdown or overexpression, and exploring their therapeutic implications through pathway-specific inhibitors. *ZWINT*, a kinetochore-associated protein, plays a pivotal role in chromosome segregation and mitotic progression. Its overexpression has been linked to tumor aggressiveness in prostate and hepatocellular carcinomas (32, 33). Our findings suggest that ZWINT may contribute to the metastatic potential of ACC by regulating mitotic progression, although further validation is needed to confirm its precise role in ACC metastasis. *CDK1*, a master regulator of the cell cycle, is frequently overexpressed in metastatic cervical, breast, and neuroblastoma cancers, where it drives G2/M transition and tumor proliferation (34–36). *CDK1* interacts with cyclins such as *CCNB1* and *CCNA2* to form complexes that regulate cell cycle progression and genomic stability. CDK1 was identified as an independent prognostic factor for poor survival in ACC patients, highlighting its critical role in driving metastatic progression. Targeting CDK1 with specific inhibitors may offer a promising therapeutic strategy for metastatic ACC. *CCNA2* is overexpressed in metastatic breast, lung, and bladder cancers (37, 38), while *CCNB1* contributes to metastasis in cervical, breast, and ovarian cancers (39, 40). These interactions reinforce the interconnected roles of these hub genes in cancer metastasis, including ACC. *TYM*S, involved in nucleotide biosynthesis and DNA repair, is overexpressed in metastatic breast, lung adenocarcinoma, and colorectal cancers (41–43). Its elevated expression has been linked to drug resistance, particularly in fluoropyrimidine-based chemotherapy, highlighting its dual role in metastasis and therapy resistance. *TPX2*, a microtubule-associated protein, is overexpressed in metastatic hepatocellular carcinoma and clear cell renal cell carcinoma (44, 45), underscoring its potential as both a biomarker and therapeutic target in aggressive tumors. These findings reinforce our hypothesis that these hub genes contribute critically to the metastatic progression of ACC.

A recent study by Behan et al. applied CRISPR-Cas9 screening across 324 cancer cell lines, identifying prioritized therapeutic targets (46). Among our seven hub genes, *TYMS* was categorized as a Group 1 priority target (targets with approved drugs), *TPX2* and *CCNA2* as Group 2 targets (potentially tractable but lacking approved drugs), and *ZWINT* as a Group 3 target (promising but with limited tractability data). These findings suggest that several of our identified hub genes are strong candidates for therapeutic targeting in metastatic ACC. Future directions should aim to validate these findings through experimental studies, particularly by assessing the mRNA and protein expression levels of these hub genes in tumor tissues and cell lines from diverse populations, including Chinese cohorts. Such validation is critical for determining their relevance across ethnic groups and for confirming their potential as diagnostic biomarkers and therapeutic targets. Moreover, functional studies (e.g., knockdown and overexpression experiments) will provide deeper insights into the mechanisms by which these genes contribute to ACC metastasis and drug resistance.

Our use of the DGIdb further revealed several drug candidates for these hub genes, including antibiotics, hormones, NSAIDs, and TKIs. For example, *TYMS* can be targeted by trimethoprim and dexamethasone, while *BIRC5* is targeted by plicamycin and sulindac. With manageable side effects, these drugs might offer long-term treatment options for metastatic ACC if proven effective, especially those targeting *TYMS* as a Group 1 candidate. Plicamycin, in particular, has shown antitumor effects in ACC cell lines NCI-H295R, BD140A, and SW-13 (47). Additionally, TKIs such as lapatinib, which have shown efficacy in other cancers, could potentially benefit metastatic ACC patients (48). Further studies to assess these drugs’ effectiveness in ACC models are warranted.

To our knowledge, this is the first study using WGCNA to investigate metastatic ACC. By identifying and verifying seven hub genes, this study provides insights into potential diagnostic biomarkers and therapeutic targets for ACC metastasis. We also identified several potential drugs with manageable side effects that may represent viable long-term treatment options. However, our study has limitations: due to the rarity of ACC, the availability of external validation cohorts and tumor specimens is restricted, which limits the immediate feasibility of performing *in vitro* experiments or animal model testing of the identified drugs. These validations are necessary to confirm the generalizability and reliability of our findings. We are actively working on obtaining additional samples and expanding our research cohort for future validation efforts. These validations are a key component of our ongoing and future research, which we plan to complete as part of our next steps. Future research should aim to validate these findings through large-scale external cohorts and functional assays in ACC cell lines or animal models. These studies will help assess the efficacy of the potential therapeutic agents identified in this study and further explore their role in improving survival outcomes for metastatic ACC. Moreover, the investigation of biological roles of these hub genes in ACC metastasis, especially through gene knockdown or overexpression studies, will be crucial in understanding their contributions to tumor progression and drug resistance. Large-scale validation studies across diverse populations are also necessary to assess the reliability of these hub genes as diagnostic biomarkers and therapeutic targets. The identified hub genes, including TYMS and BIRC5, may serve as potential candidates for clinical trials aimed at evaluating targeted therapies to improve metastatic ACC treatment outcomes.

## Conclusion

We applied WGCNA to RNA-seq data of 73 ACC cases from TCGA database and selected the most significant module concerned with metastasis. With a series of selections, we identified seven hub genes: *ZWINT*, *CDK1*, *BIRC5*, *CCNA2*, *CCNB1*, *TYMS* and *TPX2*. These seven hub genes may play a key role in ACC metastasis and may serve as promising biomarkers for metastatic ACC. Targeting drugs of them may serve as potential prevention or treatment options for metastasis ACC with tolerable side-effects. Our results may contribute to the exploration of the further and more in-depth research of metastasis ACC.

## Data Availability

The original contributions presented in the study are included in the article/**Supplementary Material**. Further inquiries can be directed to the corresponding authors.

## Supplementary material

Supplementary Table 1 PPI network of the selected module.

Supplementary Table 2 DEGs of metastasis group compared with non-metastasis group (FDR < 0.05, Fold Change > 2.0).

Supplementary Table 3 The significantly enriched GO-BP terms and KEGG pathways terms of the selected module and DEGs (*P*<0.05).

Supplementary Table 4 GSEA enriched terms of the seven hub genes.
